# Sing4Health: protocol of a randomized controlled trial of the effects of a singing group intervention on the well-being, cognitive function and health of older adults

**DOI:** 10.1186/s12877-020-01686-6

**Published:** 2020-09-18

**Authors:** Iolanda Costa Galinha, Manuel Farinha, Maria Luísa Lima, António Labisa Palmeira

**Affiliations:** 1 Centro de Investigação em Psicologia (CIP), da Universidade Autónoma de Lisboa / Universidade do Algarve, CIS-ISTE-IUL; APPSYCI, Rua de Santa Marta, 47, 3° (Room, 304), 1169-023 Lisbon, Portugal; 2 Centro de Investigação em Psicologia (CIP), da Universidade Autónoma de Lisboa / Universidade do Algarve from Rua de Santa Marta, 47, 3° (Room, 304), 1169-023 Lisbon, Portugal; 3 ISCTE CIS IUL, Av. das Forças Armadas, ISCTE-IUL building, 2w17 Room, 1649-026 Lisbon, Portugal; 4 Universidade de Lisboa & Universidade Lusófona de Humanidades e Tecnologias, Campo Grande, 376, 1749-024 Lisbon, Portugal

**Keywords:** Singing groups, Intervention, Randomized controlled trial, RCT, Cognitive function, Health indicators, Well-being, Older adults, Mediation analyses

## Abstract

**Background:**

Singing is a multimodal activity that requires physical, cognitive and psychosocial performance, with benefits to various domains of well-being and health in older adults. In recent years, research has increasingly studied group singing as an important cost-effective intervention to promote active and healthy aging. However, the specific factors responsible for these benefits need further experimental support, as most studies do not allow for causal inferences. This study responds to the need for further randomized controlled trials (RCT), with follow-up measurement, on the benefits of group singing in older adults from a low socioeconomic background. Also, while most studies often focus on specific outcome measure dimensions, in this study, the conjoint effect of several physical, psychosocial, psychoemotional and cognitive dimensions are analyzed, testing mediation effects of psychosocial and psychoemotional variables on the well-being and health of the participants.

**Methods:**

We implement and measure the effects of a singing group program for older adults, with an RCT crossover design study, in a natural context, before and after the intervention and in a follow-up, 6 months after the intervention.

**Participants:**

140 retired older adults (> 60 years) users of a social support institution, will be invited to participate in a singing group program and randomly allocated to an experimental (*n* = 70) and a control (*n* = 70) group, which will enroll in the regular activities proposed by the institution. The intervention consists of 34 bi-weekly group singing sessions, of 2 h each, for 4 months. Measures on social and emotional well-being, cognitive function, and health indicators (e.g., blood pressure, glycemia, cholesterol, c-reactive protein, sedimentation rate, respiratory function, body balance, sleep quality, medication intake, and health services attendance) will be collected. Interviews will be conducted on the motivation and perceived benefits of participation.

**Discussion:**

Significant improvements are expected in the outcome measures in the experimental group after the intervention, validating singing groups as a cost-effective intervention for healthy aging. Psychosocial and psychoemotional variables are expected to be mediators of the effects of the program in the cognitive function, well-being and health of the participants.

**Trial registration:**

NCT03985917. Registered 14th June 2019 (retrospectively registered).

## Background

Current demographic trends require validating and implementing cost-effective intervention community programs to promote physical activity, cognitive functioning, social participation, and well-being among older adults [1–3], particularly those from socioeconomically disadvantaged groups [4].

Group singing is a culturally universal activity, with potential health benefits for seniors [5]. It involves a combination of cognitive, psychosocial and physical components associated with multiple health and well-being improvements [6–8], constituting a cost-effective intervention strategy [6]. However, it is not clear what factors are responsible for these benefits, nor whether they can be causally attributed to the singing or biased self-selection of the participants. There is a lack of experimental studies capable of answering these questions [4, 9]. There is a need to expand the body of research to clarify the feasibility and effectiveness of group singing on a range of older adult physical and cognitive health outcomes [10].

### Psychoemotional aspects

Group singing has been shown to have an important impact on psychoemotional variables, stimulating emotional expression, pleasure, fun, relaxation and imagination [11]. It also fosters a sense of meaning to life, self-awareness and self-esteem [12]. In a randomized controlled trial (RCT) study with healthy older adults (*N* = 258), Coulton et al. (2015) analyzed the effects of participating in a community singing group for 3 months and a follow-up 6 months after randomization and found that participating had positive short and long-term effects on mental health, in terms of quality of life, anxiety and depression [6]. In a second RCT study with older adults from day care centers (*N* = 26), Pires, Galinha, and Herédia (2017) found that a one-month short program of group singing significantly reduced negative affect after the intervention. The positive effects, however, did not sustain until the follow-up, 8 weeks after the intervention [13]. Both RCT studies included mixed methods and the qualitative data corroborated the quantitative results. Participants reported benefits in emotional well-being along with social and physical benefits [6, 13, 14]. Mohammadi, Tanaze and Moradi (2011) in a third RCT study, with a music therapy intervention for elderly in the community (*N* = 19), that also included a group singing component, also observed a significant decrease in anxiety, stress and depression levels [15].

Other non-experimental studies also found associations between group singing and psychoemotional variables. For example, in a longitudinal study that included 633 individuals who participated in choir singing groups, the quality of life was improved, but it was difficult to recognize the direct effects of singing on the well-being [12]. Two cross-sectional studies of choir singing, were able to find a significant relation between the perceived benefits of choir singing and better quality of life on the psychological, social relations and environmental domain dimensions [16] and a better quality of life in terms of their physical health than a comparison group - even when involved in other hobbies - the singing group participants reported significantly higher overall quality of life [17]. Both singing and music listening were able to change the mood of participants immediately after a short (half an hour) session and some of the effects were visible in mood levels a week later. Although the effects were more robust for the singing group, no significant differences were found in comparison with the music listening group [18].

The present study contributes with new results on the causal effects of singing groups with old older adults from low socioeconomic backgrounds. Psychoemotional benefits on positive and negative affect, anxiety, depression and stress, self-esteem, global life satisfaction and quality of life with psychological health will be tested. Importantly, controlled causal effects of singing on some of these variables were not found in the literature. The study also allows for a conjoint analysis of benefits to the psychoemotional domain related with the other social, cognitive, and biological domains. Finally, the study is innovative in analyzing mediation effects of the psychoemotional and psychosocial variables in the effects of group singing on other domains, such as cognitive and biological health variables.

### Psychosocial aspects

Group singing is a leisure interpersonal activity with common goals, favoring empathy and social integration [15]. A few not controlled studies analyzed the impact of singing groups on psychosocial variables. Karp et al. (2006), in a longitudinal study, found that social activities have more impact when they involve a combination of psychosocial and physical components than when there is only one component, which is the case of group singing [19]. In the qualitative data of the Pires et al. (2017) study, social benefits were one of the most frequently reported advantages from participating in the singing group in the interviews, in terms of interpersonal interactions, levels of social support among seniors and sharing of positive emotions [13]. Stewart and Lonsdale (2016) compared choir singing with both solo singing and another leisure group activity (sports). They found that choir singers and team sport players reported higher levels of well-being than solo singers; moreover, the members of the choir experienced a stronger sense of being part of a meaningful group than the members of a sports group [8]. Thus, belonging to a group with an identity, to which individuals feel attached, may favor feelings of inclusion and social support. Participating in a choir, with vocal harmonizing and social interactions during rehearsals may favor interdependence and connection between the group members. Furthermore, increased social integration of the participants (higher perceived social support, lower feelings of loneliness, greater sense of social integration and identification with the group) may have positive consequences on their physical health [20–22].

The present study contributes with new results of the effects of singing on loneliness, identification with the social group, social well-being and quality of life (related with social relationships, with past, present, and future activities, and with social participation). The study will also test mediation effects of psychosocial variables of the effects of group singing program (such as group identification and loneliness) on the cognitive, mental and physical health variables of the participants.

### Cognitive functioning aspects

Group singing activity stimulates cognitive processes by focusing attention on the music, the teacher’s orientation, and the interaction with the other singers, memorizing lyrics, pitch and rhythm, factors which are known to stimulate executive, attention, verbal learning and memory functions [4]. Musical activities involve an extensive stimulation of the brain (nervous and neuroendocrine systems, temporal, frontal, parietal, cerebellar and limbic areas), capable of inducing long-term neuroplasticity and improving emotional and cognitive functions [23]. A few studies tested the impact of singing groups on the cognitive functioning of older adults with severe cognitive deficits. An RCT study by Särkämö (2014) with a dyadic intervention component, included people with dementia and their caregivers (*N* = 89), who acted as a coach, in two experimental conditions for a period of 10 weeks. Results showed that singing and listening to music were associated with improvements to remote episodic memory, attention, executive and general cognitive functions, while singing, specifically, revealed improvements in short-term and working memory. Benefits of the intervention, however, were more evident in individuals with early dementia [24]. Another study that compared singing activity and listening to music in older adults with dementia showed that singers reported significantly higher subjective well-being and cognitive ability improvements than listeners [25]. In another RCT study with older adults diagnosed with Alzheimers (*N* = 10), in which the music therapy group participated in weekly singing sessions for a period of 6 months, showed a significant decrease in the completion time of the Raven Matrix test and also in the results of the neuropsychiatric inventory between the pre and post-test intervention period of 6 months [26]. Functional magnetic resonance imaging revealed increased activity in the right angular gyrus and left lingual gyrus during participation in musical activity.

Fewer studies with singing groups included older adults with intact cognitive function or low cognitive deficits. Fu and collaborators (2018), in a quasi-experimental design, with 49 participants with no self-report of dementia, found that a singing group program of 75 min weekly sessions, for 12 weeks, with deep breathing training and song learning showed significant in promoting memory, language, speech information processing, and executive functions [10]. Clements-Cortes (2014) also included two small groups of 16 participants, one with intact cognitive function and another with dementia, in a chorus intervention for 10 to 16 sessions, yielding significant benefits in positive emotions and perceived pain, although, with no reports on cognitive benefits [27].

In sum, more RCTs are needed to test specific effects of choir singing on the cognitive functions of older adults with varying levels of cognitive function. Our study aims to contribute with causal effect results of a singing group program in terms of the general cognitive functions. We expect that weekly exercises of attention focusing on the lyrics, music and silent pauses, teachers’ instructions, and voices harmonizing with the group, and also with more social contacts and communication may work as a stimulation of the brain processes and structures. This stimulation can produce improvements in executive function, short-term memory, language, attention, working memory, verbal memory, and speed processing. The study also tests the hypothesis that cognitive function acts as a moderator of the effects of group singing on the physical and mental health of the participants.

### Health aspects

Aging is associated with declines in health, such as respiratory function, weaker lower limb strength and balance, cardiovascular disease, diabetes, high blood pressure, among others. In part, the individual’s physical decline is explained by a sedentary lifestyle and may be decelerated by keeping up physical activity and social participation. Participation in singing groups presupposes a regular physical and social activity, which requires changes to daily routines, increased physical activity, respiratory activity, mobility and longer periods standing up. Only a few experimental or quasi-experimental studies have provided evidence of measurable health benefits of a singing group activity [28]. In a quasi-experimental study, following participation in a choir program for 12 months and 24 months (Cohen et al. 2006, 2007), the intervention group reported a higher overall rating of physical health, fewer doctor’s visits, less medication use, fewer instances of falls, and fewer other health problems than the comparison group [29, 30]. However, due to methodologic and statistical analysis flaws of this study, analyzed in subsequent reviews of the literature [9, 31], these results should be considered very cautiously, and the hypotheses retested in future studies.

#### Physical activity and sedentary behavior

A recent study analyzing two large groups of older adults in the US and in the UK, using actigraphy to objectively measure these variables, found that about two thirds of awake time is devoted to sedentary activities, while only 2.5 up to 4.1% of subjects attained the 30 min/day moderate to vigorous physical activity recommendations [32].

#### Sleep quality

Sleep problems are increasingly common as one ages, and it is known that sleep duration is associated with all-cause mortality. There is an inverted U relationship between sleep duration and health problems, in that both short and long sleepers (< 6 h and > 8 h night, respectively) show a higher risk of developing health issues [33]. Both physical activity and sleep quality may be measured through actigraphy. However, none of these indicators have yet been analyzed as outcome measures of singing interventions in older adults.

#### Respiratory function

The aging process leads to a decline in the respiratory system resulting in changes to lung structure, the shape of the chest wall, thoracic muscles, and pulmonary vascular circulation [34]. Participating in group-singing activities, requires effort in breath management, including breathing support and control that may promote pulmonary health [10]. This result was found by Bonilha et al. (2009) in an RCT with 78 participants in a 6-month weekly singing program, improving lung function as regards inspiratory capacity, decreases in expiratory reserve volume and better maximal expiratory pressures, in patients with chronic obstructive pulmonary disease, following the singing therapy program [35]. The previously mentioned quasi-experimental study of Fu and collaborators (2018) also found respiratory muscle strength improvements in older adults associated with the singing group intervention [10]**.** Another study with a singing therapy intervention showed improvements in pulmonary function, through the reduction of lung hyperinflation, and an increase in arterial blood oxygen saturation. Most participants also reported an increase in exercise capacity and the ability to control symptoms by breathing techniques acquired during the singing lessons, as well as a reduction in anxiety and a perceived improvement in quality of life [36]. Therefore, the learning of breathing techniques and the exercise of breathing control required in singing may be considered an enjoyable lung health promotion activity. Literature reviews on the efficacy of singing programs for lung health, however, found inconsistency in the available results and difficulty in comparing studies due to the diversity of methods and designs [37]. A few studies that evidenced benefits of singing for people with obstructive pulmonary disease showed limited validity due to small sample sizes and no evidence of long-term effects [38]. The findings suggest that the use of singing group therapy may improve older adults’ lung function, as a potential defense against functional decline, however, studies are scarce and more RCT studies are needed to confirm causal and long-term effects.

#### Blood biomarkers

The increase in physical activity and respiratory function stimulated by a weekly singing program may also have an impact on blood biomarkers, such as blood pressure, blood cholesterol, blood glucose, inflammatory and immune biomarkers. For example, Beck et al. (2000) following a naturalistic pre and post-test design during rehearsals and in a public performance found that the mean levels of secretory immunoglobulin A increased significantly - 150% during rehearsals and 240% during the performance. Conversely, cortisol concentrations decreased significantly on average 30% during rehearsals and increased 37% during performance, suggesting that singing activity activates the immune system [39]. Similar results were found by Kreutz et al. (2005), after two rehearsals, where positive affect and secretory immunoglobulin A (S-IgA) increased and negative affect decreased. In another study, saliva samples were collected to assess cortisol and alpha amylase. The results revealed that singing activity had positive influences on affect and social connectedness, however biomarker changes were not significant across all experiments and the unexpected absence of biological effects warrants further investigation [40]. Measuring and analyzing immunity biomarkers, however, is demanding in resources, as proposed by Ryan et al. (2016) and most studies do not follow the right protocol, resulting in lower validity of the results. In this study, due to limited resources, we limited the analysis to inflammatory blood biomarkers [41]. Other biomarkers, such as blood pressure and heart rate, were measured by Valentine and Evans (2001), before and 30 min after a solo singing activity, choir singing and a swimming session among young adults. Engaging in these activities reduced tense arousal and increased energetic arousal, positive hedonic tone and heart rate. The effects were higher in the case of swimming than for singing, with little difference between choir and solo singing [42].

Cardiovascular disease is one of the largest health problems and the leading cause of death among older adults. Intervention and policy approaches are shifting focus to multiple behavior change strategies, targeting problems with common factors. Singing is associated with benefits for health and well-being across the lifespan, with observed changes in the perception of illness, mood, anxiety, and blood pressure levels. However, studies are needed to determine whether such changes are caused specifically by the activity of singing [43]. Very little is known about the effects of enrollment in singing groups on the health biomarkers of participants, such as blood sugar, cholesterol, inflammatory indicators, and blood pressure. In this study, the effects of group singing on a set of health biomarkers are analyzed, expecting to identify wider therapeutic benefits of group singing interventions.

#### Perceived health

From the associations previously reported between participating in group singing and higher well-being and physical and sensory activity, it is possible to hypothesize an impact, not only on the physical health, but also on the perceived health of the participants, such as health-related quality of life (Guralnik et al., 1994) [44], the perception of pain, and related behavioral variables such as the demand for health services and medication intake (Cohen et al., 2006, 2007) [29, 30]. However, some of these results require replication with higher quality methods [9, 31].

#### Perceived benefits of singing

In qualitative studies on the perceptions of older adults’ participation in singing groups, social support and increased interaction between participants, the expression of positive emotions and knowledge acquisition are reported [13], as well as the emotional, physical and mental well-being and learning new things [45]. In this study, interviews will be collected on the perceived motivations, expectations and benefits of those participating in the program, allowing to substantiate the quantitative and qualitative results of the study and thus achieving a higher understanding of the results.

As previously stated, the scientific literature on singing group interventions has demonstrated benefits on the well-being, quality of life, lung function, depression, anxiety, and stress symptoms of older adult participants [28]. However, literature reviews also highlight the lack of RCTs with blinding of conditions and follow-up collection, and limitations of the studies including biased selection of participants, small sample sizes, and selective reporting of outcomes, resulting in the weak validity of some of the results. More RCTs are needed to further validate the benefits of singing groups on the emotional and social well-being, cognitive function and health of the participants (Clark & ​​Harding, 2012; Clift et al., 2016; Dingle et al., 2019) [28, 46, 47].

In this study we aim to overcome some of the gaps in the literature, implementing a pragmatic RCT study, with a 6 months’ follow-up and single-blinded data collection. The protocol of the study was pre-registered to avoid selective outcome reporting. The study enrolls a sample of 140 participants, including young and old older adults from disadvantaged socioeconomic groups, not previously involved in singing groups. This is also a mixed method study with quantitative and qualitative data analysis. The study tests the causal effects of a singing group program on the psycho-emotional, psychosocial, cognitive, and health biomarkers variables, assessed through a series of self-reports, physical trace records, and cognitive and physical performance tests. The study will also qualitatively analyze the participants’ perceptions of their motivations and of the benefits of participating in the program, allowing us to establish relationships with the quantitative results obtained through the RCT design of the study.

**Fig. 1:**
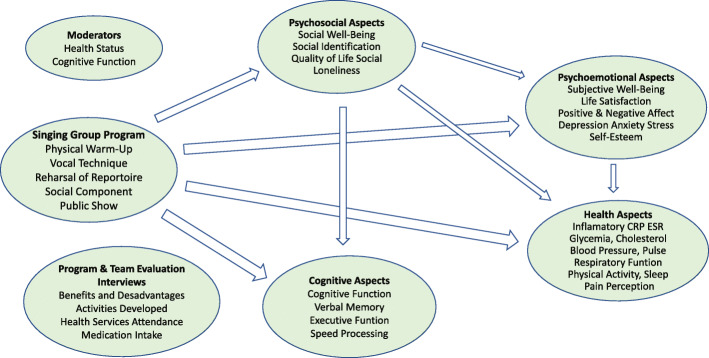
Biopsychosocial Model of the study depicting the hypotheses of causal effects (Psychosocial, Psychoemotional, Health and Cognitive Outcomes) and mediation effects (Psychosocial, Psychoemotional) of the Singing Program Components (Physical, Cognitive, Social and Emotional)

Our hypotheses are that participating in group singing will significantly improve the emotional and social well-being, cognitive function, and the general health of the participants after the program, and significantly different to the control group. We also hypothesize that psychoemotional variables (e.g. self-esteem; depression, anxiety and stress), and psychosocial variables (e.g. social identification; loneliness), will mediate the effects of the singing group program on cognitive function (e.g. executive function, verbal memory), well-being and health (e.g. blood biomarkers, respiratory and motor functions) of the participants (see Fig. 1).

## Method

### Study design

**Fig. 2:**
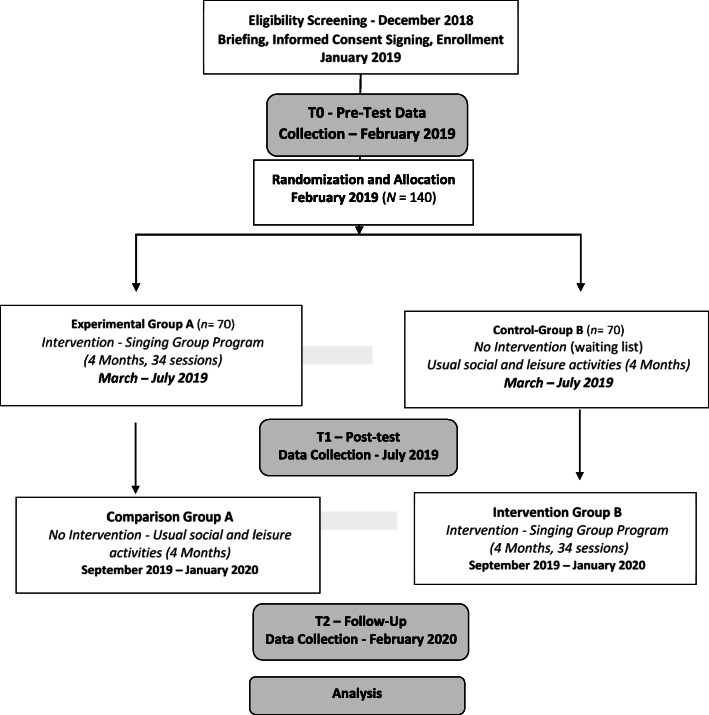
Randomized Controlled Trial Crossover design of the study

The present study is a RCT with a crossover design with two conditions (experimental and control), where the participants will be randomly allocated to the two groups. Participants in the experimental group (EG) will take part in the singing group activity, while the control group will be involved in the usual social and artistic activities provided by the social support institution. Data collection will take place prior and after the singing group program and follow-up for the experimental group (6 months after the singing program has finished). The control group will participate in the program after the experimental group and post-test data has been collected from both groups. Semi-structured interviews on the motivations and perceived benefits of participating in the singing group will also be collected on the three times of data collection (see Fig. 2). In this study, we have an immediate intervention group (IG) and waiting-list control group (WLG). After the second assessment (T1), individuals in the control group will crossover to the intervention arm and participate in the singing group program. Thus, this study comprises a randomized experimental phase (T0 to T1 with a within- and a between-groups comparison) and a non-randomized quasi-experimental follow-up phase (T1 to T2 within groups comparison). We opted not to create a specific alternative activity for the control group individuals. Participants are instructed to maintain their daily usual social and leisure activities during the same period. All activities and the duration of the activities developed by the participants during the intervention program and the washout period will be registered for control.

### Participants

Senior adults, users of the various services of the Santa Casa da Misericordia de Lisboa and Santa Casa da Misericordia de Almada (day-care centers, nursing homes and home care) will be invited by the staff of the institution to participate in an intervention and research program on singing groups. The Santa Casa da Misericordia (SCM) is a Portuguese social support institution whose mission is to improve the quality of life of the less privileged. The inclusion criteria for participants is being 60 years old or older, retired, accepting the invitation to participate in the program, and not having participated in other structured intervention programs in the previous 4 months. Exclusion criteria is having a severe diagnosed impairment that is an impediment for participating in the singing group activity (e.g. severe auditory, visual or mobility impairments). Sample Size. One hundred and forty participants will be invited to participate in the singing program and randomly assigned to an experimental (*n* = 70) or a waiting-list control (*n* = 70) group. A post hoc power analysis shows that this sample will be able to detect a large effect size (n2 = 0.14) with an 88% power. A dropout of 15 participants per group will reduce the study power to 79%. In order to promote adequate enrolment, transport will be provided for participants to attend the intervention sessions. To promote retention and complete follow up, the intervention team and the social support institution promote two social events related to the intervention program. An event for welcoming the second intervention group by the first intervention group will take place, with video presentations and singing together, and a final show with the participation of both intervention groups, after follow-up measurement.

### Procedures and randomization

**Fig. 3:**
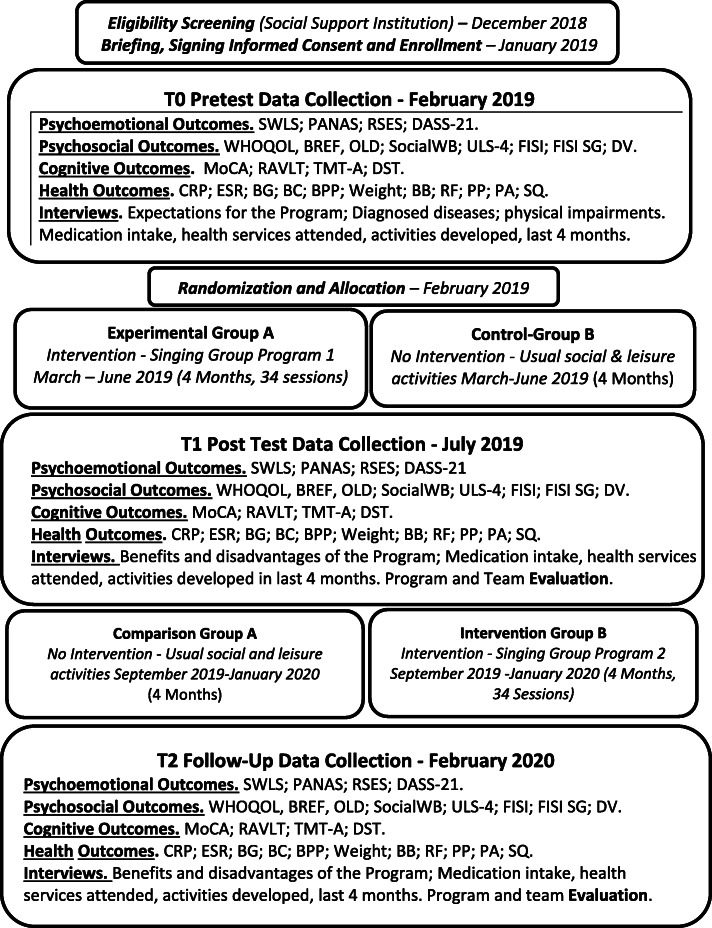
Timeline of the Schedule of Enrollment, Interventions and Assessments

The social support institutions’ that are partners of the study will select potential participants, according to the eligibility criteria of the study and their records of the participants. All eligible older adults will be invited to participate in a singing group program, included in a scientific research study. A briefing will be organized by the research and intervention teams to answer the doubts of the potential participants and to explain the content of the informed consent. After the signing of the informed consent, pre-test measures will be collected, and randomization will be performed. The scientific coordinators of the study will set a numerical ordering of the list of participants and, using the SPSS randomization tool, will split up the participants into two equal groups. The social support institution will assign the selected participants to the intervention program. The participants, singing group facilitators and researchers conducting the study cannot be blinded to allocation. However, the collection of the data, namely, cognitive, psychosocial, motor and physiologic outcome measures will be assessed by independent researchers, blinded to group allocation (see Fig. 3).

Data collection will take place for 2 weeks, 10 weekdays, from 10 to 15 participants per day. It will start at 8 am, while participants are fasting, for: I) biomarkers, respiratory function and balance data collection, followed by a buffet breakfast; II) cognitive measurement, followed by a 15 to 20 min break; and IV) psychosocial measurement (questionnaires followed by semi-structured interviews). Every step of data collection will be performed by a different researcher.

### The intervention program

The experimental group will participate in a singing group program that includes several components: (1) relaxation exercises and vocal warm-up; (2) vocal technique component; (3) rehearsal of repertoire component; (4) social component; (5) creation and presentation of a final performance; and (6) assessment of each participant’s performance. Duration: The program is developed over 34 sessions, for 4 months, 2 days a week, 2 h of group singing, with a 20 min break for socialization. Venue: The rehearsals take place in local theatres, outside the day care centers, improving the mobility of the seniors and the contact with the community. A strategy to improve adherence to the intervention program consists in including public figures, well-known singers of this target group, that invited the participants to program, participated in rehearsals and in the final performance (see Table 1).

**Table 1:** Singing Group Intervention Program Sing4Health Description (TIDieR: Template for Intervention Description and Replication)

**BRIEF NAME:** Sing4Health - Sing for your Health, Singing Groups Intervention Programme for Older Adults	
**WHY:**	
Previous literature has suggested benefits of singing groups for the well-being, cognitive function and health of older adults. Thus, the objective of this program is to invite a group of older adults to participate in a singing group intervention, to learn a set of exercises and vocal techniques, and collaborate in the creation of a singing show for the general public. The expected benefits of this social and artistic group activity are improvements in the emotional and social well-being, cognitive functioning and general health of the participants.	
**WHAT:**	
**I Materials**:	
One rehearsal room with the capacity for 35 seated individuals and an additional 40 square meters for exercises and activities, one chair for each participant, one piano, one file with lyrics, one bottle of water for each participant, one separate coffee break room, transport for the older adults.	
**II Components and Procedures of the Group Singing Program:**	
**1. Component of physical exercises for relaxation and vocal warm up -** The rehearsals start with 15 mins of relaxation exercises (adapted to the age and physical mobility of the participants) and vocal warm-up exercises. Physical aspects of choral singing are exercised and discussed to promote body awareness, a healthy body posture and understanding of effective breathing during singing. The singing sessions include a combination of sitting and standing up, the use of movements to follow the rhythm and moving to different parts of the room to sing. **Provided by** the intervention team (pianist, maestro) with the support of older adults’ assistants.	
**2. Vocal technique -** The program aims to provide a complete group singing experience for seniors without musical experience, teaching fundamental aspects of singing, sound, voice mechanics and group singing practice. Participants learn correct and healthy vocal production skills, using breathing techniques and listening skills. Participants learn to sing in different parts or voices, which are taught by hearing and repetition. They learn to listen to each other and to synchronize with each other. **Provided by** the intervention team (pianist, maestro) with the support of older adults’ assistants.	
**3. Rehearsal of repertoire –** Sessions involve memorization and interpretation of songs and lyrics using different strategies (learning to listen to separate parts, singing lyrics or musical notation, call and response methods), reviewing previously learned songs and learning to harmonize the individual voices with the rest of the group. The repertoire is proposed by both the intervention team and the participants and selected by the whole group after discussion. The repertoire should also be chosen according to the responsiveness and meaning for the participants and with cultural interest for the community in general. Provided by the intervention team (pianist, maestro, invited artist) with the support of older adults’ assistants.	
**4. Social Component - Break for Socializing and Snacks -** Each session has a break of about 20 min for a snack and socialization among the participants. During the break and at the end of each rehearsal, a social interaction is promoted in which group members share emotions, ideas and expectations about their participation in the program. The sessions also include work oriented towards a common goal, group dynamics exercises and discussions about the meaning of the songs and their cultural history. **Provided by** the intervention team (pianist, maestro) with the support of older adults’ assistants.	
**5. Creation and Presentation of a Public Performance** - The sessions are organized in order to prepare a final performance for the general public. A singer is invited to sing with the group. Families of the participants, older adults from day care centres and the public in general are invited. The participants will benefit from make-up and hair styling before the show. **Provided by** the intervention team (pianist, maestro, and invited artists) make-up artists, supported by the older adults’ assistants.	
**6. Evaluation –** Participants’ tuning will be assessed at the beginning and at the end of the program. To better adjust objectives, the intervention team will monitor the evolution of each participant, in terms of the skills acquired, record of attendance, and achievement of objectives. The quality of the programme and intervention team will be evaluated by the participants by a questionnaire. **Provided by** the intervention team (pianist, maestro), assistant researchers and supported by older adults’ assistants.	
**7. Audio-visual Recoding of the Programme –** A media audio-visual team will collect images from the development of sessions and reports from the participants and the final show, to produce a documentary (20 to 30 min) and two short videos, for the dissemination of the program. The team will be integrated in the singing groups from the beginning. **Provide by** the media team.	
**Team expertise:**	
**Maestro** – Master in Music specialising in Choral Direction by the *Escola Superior de Música de Lisboa*; **Maestro** (Substitute) – Degree in Music in the Community by the *Escola Superior de Educação e Música de Lisboa*; **Pianist** – Master in Jazz Performance by the Royal Welsh College of Music and Drama; **Artistic Director and Invited Artist** – Professional Singer, Musicals’ Performer and Master in Psychology by the Universidade Autónoma de Lisboa; **Invited Artist** – Tenor, Resident Opera Singer of *Teatro Nacional de São Carlos*; **Older Adults’ Assistant** – Degree in Social Education, specialising in Rehabilitation. **Older Adults’ Caretakers** – Professional caretakers from the social support institution. **Training for the Program:** A workshop of two sessions will be provided for all members of the intervention team on the components and goals of the intervention program and on working with the elderly.	
**WHERE**	
Sessions will be conducted in the rehearsal rooms of local theatres in Lisboa and Almada. The performance will take place in public theatres.	
**WHEN and HOW MUCH**	
**Singing Program Duration:** 34 sessions, for about 4 months, 2 days a week, each with 2 h of group singing, with a 20-min break for socializing. **Participants:** Four groups of 30–35 participants.	

While the experimental group is participating in the intervention program, the control group will take part in the usual activities proposed by the day care centers (SCM), and these activities will be registered. After the experimental group finishes the program, the control group will enroll the exact same program. In time three of data collection, the same measures will be collected, representing a follow up for the participants of the first intervention group and a post-test for the second intervention group. All the financial costs of the intervention will be registered and information on all appointments attended in health care services and medication intake by the participants, during the period of implementation of the program, will be collected from both the social care institution and the participants. This will make it possible to calculate the difference between the costs of the program and the expected benefits of the program: for example, if significantly less health care services were used during the intervention periods and its estimated costs (e.g., medical appointments and hospital stays) were therefore lower.

### Ethical procedures

This project follows the international criteria of ethical requirements for studies with vulnerable human participants and for the protection of personal and sensitive data. The research project was approved by an independent Ethics Committee. The terms of participation of the institutions will be subject to written agreement. A debriefing session will be provided by the intervention and researchers team to potentially interested participants, that will sign an informed consent obtained by the social support institution staff, detailing the terms and conditions of participating in the singing program and in the study (available upon request to the authors). Blood samples will be collected, treated and stored for health assessment purposes, following the standards set out in European directives, through the contract of a certified laboratory.

The personal, psychological and health data of the participants will be stored separately, in files password-protected to ensure the confidentiality of the data. During data collection, only the participant number in the survey protocol will be recorded. All information that can identify participants (e.g., name, address, contacts) will be assigned a code number and recorded in a separate file. In a third file, the participant’s number will be associated with the personal information code. The ability to link indirect identifiers with names and addresses remains, but only the coordinator researchers will have access to that link, stored securely and password protected in the study site. If individuals wish to access or modify their registered personal information, they may do so at any time by contacting the scientific coordinators of the study. Data will be archived and kept confidential for at least 10 years after the publication of the results. Data monitoring committee is composed by the research coordinators of the study, independent from the sponsor and of any competing interests. None of the authors of the study have any competing interests. The final trial dataset will not be shared with researchers outside the project team. However, the database will be readily accessible for reviewers in a way that preserves the anonymity of participants.

The order of authorship of the scientific outputs is defined by the scientific contribution in the study, namely, the conception, implementation, writing and scientific supervision, first authored by the PI (Principal Investigator), followed by the co-PI (co-Principal Investigator) and other researchers’ contributors. Important modifications to the protocol will be reported to relevant parties, namely, public protocol registry (NCT03985917) and ethics committee for approval. Research will also follow the standards of scientific integrity and transparency to ensure ethical criteria in the production and dissemination of scientific knowledge.

### Materials

The study aims to measure a set of biopsychosocial variables, described below, divided into four domains: psychoemotional, psychosocial, cognitive and health. All measures will be collected to measure change from baseline at 4 months (post-test) and at 10 months (follow-up).

#### Psychoemotional variables

**Life Satisfaction** will be measured by the Satisfaction with Life Scale (SWLS, Diener, Emmons, Larsen, & Griffin, 1985) validated to the Portuguese by Neto, Barros, and Barros (1990) [48, 49]. Satisfaction with life in general is assessed according to the person’s own criteria. Consists of five items, answered on a 5-point scale from: 1 ‘strongly disagree’; to 5 ‘I agree very much’.

**Positive and Negative Affect** will be measured by the PANAS - Positive and Negative Affect Schedule (Watson, Clark, & Tellegen, 1988) validated for the Portuguese by Galinha and Pais-Ribeiro (2005) [50, 51]. It consists of two scales with 10 items each, which measure the frequency of positive emotions and negative emotions (e. g, “To what extent did you feel each of the following emotions during the last days”: enthusiasm, distress, etc.) on a five points Likert scale (from 1 “nothing or very slightly”; to 5 “extremely”).

**Self-Esteem** will be measured by the Rosenberg Self-Esteem Scale (RSES, Rosenberg, 1965) validated for the Portuguese by Pechorro, Marôco, Poiares, and Vieira (2011) [52, 53]. Assesses the appreciation of self-worth and self-acceptance, with 10 items, answered on a 4-point Likert scale, from 1 ‘strongly disagree’ to 4 ‘strongly agree’.

**Depression, Anxiety and Stress** will be measured by the DASS-21 (Lovibond & Lovibond, 1995) validated for the Portuguese by Pais-Ribeiro, Honrado and Leal (2004), which consists of 21 items distributed over 3 subscales of 7 items each, related to one of the following constructs: depression, anxiety or stress [54, 55]. The response to the items, the extent to which the each sentence applies to the subject in the last 4 months is classified using a Likert 4-point scale ranging, in terms of severity or frequency, from 0 “It did not apply to me” to 3 “Applied to me most of the time”.

#### Psychosocial variables

**Quality of Life** dimensions will be measured by two instruments:

- The **World Health Organization Quality of Life – Old** (WHOQOL-OLD) from Power, Quinn, Schmidt, & The WHOQOL-OLD Group, 2005), adapted to the Portuguese by Vilar (2015) [56, 57]. The WHOQOL-OLD measures the quality of life specifically for older adults, with 28 items, measuring six dimensions. In this study the dimensions (1) sensory abilities; (2) autonomy; and (4) social participation will be measured. The items are answered in a 5-point scale, from 1 (Nothing; Very Bad; Very Unsatisfied) to 5 (Extremely; Very Good; Very Satisfied);
- The **World Health Organization Quality of Life – Bref** (WHOQOL-BREF, WHOQOL Group, 1998) adapted to the Portuguese by Vaz Serra et al. (2006) [58, 59]. The WHOQOL-BREF measures the subjective perception of quality of life. It consists of 26 items, of which two items measure global quality of life and 24 items measure four dimensions. In this study the (1) Physical health, (2) Psychological Health, and (3) Social relationships will be measured. The items are answered in a 5-point scale, from 1 (Very Bad; Very Unsatisfied; Nothing; Never) to 5 (Very Good; Very Satisfied; Extremely; Always).

**Loneliness** will be measured by the UCLA Loneliness Scale (Pocinho, Farate, & Dias, 2010) [60]. Assesses the feeling of being cut off from others, with four items, derived from the longer version of the ULS-20, answered on a 4-point scale from: 0 ‘Never’, to 4 ‘Often / Many Times’.

**Social Identification** will be measured by the Four Item Measure of Social Identification FISI (Doosje, Spears, & Ellemers, 1995) [61]. Assesses the emotional evaluation of the relationship between the self and the ingroup, with four items answered on a Likert scale, ranging from 1 ‘strongly disagree’ to 7 ‘strongly agree’.

**Social Well-Being** will be measured by the Scale of Social Well-Being (Keyes, 1998) adapted for the Portuguese by Silva (2016) [62, 63]. Assesses a multidimensional construct encompassing the dimensions: social acceptance, social coherence, social actualization, social integration and social contribution. In this study we will use a short version of 6 of the 33 items, answered on a Likert scale ranging from 1 ‘strongly disagree’ to 6 ‘strongly agree’.

#### Cognitive function variables

**Cognitive Function** will be measured by the Montreal Cognitive Assessment MoCA (Nasreddine et al., 2005) validated to the Portuguese by Simões et al. (2008) and Freitas et al. (2012) [64–66]. The test measures six cognitive functions: executive function; visuospatial skills; short-term memory; language; attention, concentration and working memory; and temporal and spatial orientation. The maximum score is 30 points, higher scores indicating a better cognitive performance.

**Short-term auditory verbal memory** will be measured by the Rey Auditory Verbal Learning Test (Schmidt, 1996), validated to the Portuguese by Cavaco et al. (2015) [67, 68]. The scale measures the rate of verbal learning, learning strategies, retroactive, and proactive interference, presence of confabulation or confusion in memory processes, retention of information, and differences between learning and retrieval. It consists of a list of 15 unrelated words repeated over five different trials, which participants are asked to repeat in five trials.

**Attention and Executive Function** will be measured by the Trail Making Test (Reitan, 1958) adapted to the Portuguese by Cavaco et al. (2013) [69, 70]. The test measures processing speed, mental flexibility, and divided attention. In this study, TMT A will be used, providing two direct scores: time to complete part A and performance errors.

**Processing Speed** will be measured by the Digit Symbol Test from the Portuguese version of WAIS-III (Wechsler, 2008) [71]. The test measures the processing speed of non-verbal information. It consists of a task of drawing figures corresponding to numbers. It also assesses cognitive and motor speed, planning ability, visual memory, visuomotor coordination, motivation to perform the task and attention.

#### Health indicators

A set of physiologic biomarkers, motor function, health and pain perception, health services attendance and medication intake will be collected.

**Biomarkers** will be collected early in the morning and fasting by a licensed laboratory: (1) **blood pressure** measures the pressure with which the blood circulates within the arteries; **pulse** measures the count of arterial pulse per minute, the number of times the heart beats per minute; (2) **body weight** is assessed by weighing the person dressed in light clothing, without shoes and while fasting; (3) **Glycemia,** the amount of glucose present in the blood, used in the diagnosis and treatment of several diseases such as diabetes mellitus or hypoglycemia; (4) **Cholesterol (total),** a fatty substance produced by the liver, present in all body cells and essential for the formation of the cell membranes, digestion of fats, production of bile, metabolism of vitamins A, D, E and K, important in the constitution of global coronary disease risk; (5) **C-reactive protein (CRP),** a protein produced in the liver that in the case of inflammatory states increases in production. Indicates an ongoing, but not specific, organism infection. It can also be high in case of neoplasia; (6) **Erythrocyte Sedimentation Rate (ESR)** it is a measure of the red blood cells’ sedimentation by micro photometry over a period of time. A blood sedimentation rate is tested to detect inflammation in the body or to follow the progress of a disease.

**Respiratory Function (air volume and air speed)** will be measured by spirometry, using a portable spirometer (Medikro Pro). The air volume test consists of a simple examination, which allows to globally evaluate how the lungs are functioning by measuring the volume of air (measured in liters with the tests FVC, FEV1 and FEV6). In addition, the Tiffeneau-Pinelli Index will be measured, which is a ratio between FVC and FEV1. The air speed test consists of a simple examination, which allows to globally evaluate how the lungs are functioning by measuring the speed of air expired (litres per second, measured with the tests PEF, FEF 25 to 75%). Data is collected by trained researchers in motricity sciences.

**Body Balance** will be measured by the unipedal stance test. This test is suggested as a viable option, specifically when several measures or time constraints are in place (Whitney, Poole, & Cass, 1998) [72]. In this test the participant is instructed to balance on one foot of their choosing for up to 20 s. The number of seconds in balance is registered as the score of this test. Data collected by trained researchers in motricity sciences.

**Physical Activity** will be measured by actigraphy. The GT3X accelerometer that will be used in this study, is capable of recording accelerations in three axes (vertical, antero-posterior, and medio-lateral). Participants will be asked to wear the accelerometer around the hip for seven consecutive days (day and night, except when bathing). The accelerometer provides a measure of the frequency, intensity, and duration of physical activity and allows for the classification of activity levels as sedentary, light, moderate and vigorous [73].

**Sleep Quality** will be measured by actigraphy. The GT3X allows for a measure of the total duration of sleep of the subject [73]. The accelerometer provides a measure of the frequency, intensity, and duration of physical activity and allows for a measure of the total duration of sleep. Participants will carry the device with them at all times during a week, except when bathing.

**Pain Perception** is measured by the Brief Pain Inventory, adapted for the Portuguese by Ferreira-Valente, Pais-Ribeiro and Jensen (2009) [74]. Assesses different types of pain, including 9 questions: a first that is optional, a second one based on pain drawing diagrams, four items about pain intensity (worst pain, least pain, average pain, pain right now), two items on pain relief or medication, and one item on pain interference, with seven sub-items (general activity, mood, walking ability, normal walk, relations with other people, sleep, and enjoyment of life). Answering options vary between 0 ‘No pain/interfered’ and 10 ‘The greatest pain possible/completely interfered’.

**Health Status, Health Services Attendance and Medication Intake** – Data on the participants’ diagnosed diseases at baseline, the number and type of health services appointments, and medication intake during the various stages of the program will be registered by the Social Care Institution and provided to the research team with the authorization of the participants.

### Musical training and sensorial abilities

**Vocal Tuning Abilities** will be measured before and after the second intervention program through a test with two exercises. Notes (task 1) and chords (task 2) are played on a piano and participants are asked to sing the notes. Vocal tuning will be measured with an electronic tuner, indicating how close the vocal harmony was from the played note. One point is assigned for each correctly sung note, with a maximum score of 5 points in task 1 and of 6 points in task 2.

### Perceived motivation, expectations and benefits of participating in the program

The study also includes semi-structured interviews on the motivations, expectations and perceived benefits of participating in the program, positive and negative aspects of the program and suggestions for future programs. Also, the control group is asked about in which activities they participated while the first intervention was developing. The same will be asked to the experimental group in the follow up (see Fig. 3). Semi-structured interviews will be performed by researchers after all the other structured questionnaires and tests have been collected. The interviews will be recorded and verbatim transcribed. The content analysis will be performed after reducing the responses to minimal units of meaning, following a categorization by thematic coding according to the objectives of the interviews described above. Two researchers will work on the analysis independently, after which they will meet to discuss the analysis. Any disagreements in the content analysis of the interviews will be resolved through consensus.

All measurements are validated for the study population language with adequate psychometric properties. Additionally, the team will perform psychometric analysis of the measures in the study sample. Assessors will be trained for data collection with guidelines for instruments and measurement procedures, and for working with older adults. Data collection forms can be obtained upon request from the authors. Data entry and coding are subject to two rounds of verification by the peer assessors and by the PI.

Statistical methods include mean comparison of outcome measures (chi-square, t-test, general linear model for repeated measures analysis and ANOVA, after statistical assumptions are verified) between the three times of data collection (pre-test, post-test after 4 months and follow-up after 10 months) and between experimental and control groups. Two experimental sub-groups will be analyzed, as two intervention groups were formed, although with the exact same conditions. Additional, structural equation modelling analysis will be performed to test regression and mediation model hypothesis. No interim analyses are planned. Intention to treat strategies, imputing last observation carried forward, may be used.

## Discussion

With this randomized controlled trial, we aim to examine the effects of participating in a singing group program on various psychoemotional, psychosocial, cognitive and health variables in older adults. Recent studies have tried to validate singing group intervention as a cost-effective strategy to promote active and healthy aging. Some results suggest the benefits of participating in singing groups for older adults in some of these variables. However, most studies lack randomized controlled study designs, not allowing establishing causal inferences of the effects of group singing interventions. Secondly, some studies analyze the benefits of participating in singing groups in people previously enrolled in these groups and whose motivation for the activity is already substantial. Additionally, most previous studies are developed with younger older adult participants and with higher social status. It is important to test the effectiveness and feasibility of singing groups interventions with young and old older adults from low socioeconomic backgrounds and minority groups.

Therefore, to overcome some of the gaps in previous studies on singing groups, we designed a randomized control trial of a singing group intervention, with follow up measurement. Participants from social support institutions, from low socioeconomic backgrounds, young and old older adults, who have not participated in any recent singing activity, are invited to enroll in a singing program.

Some of the variables included in this study have not been previously tested as outcomes of singing group programs in RCT studies, namely, blood biomarkers (inflammatory, blood glucose, blood pressure), respiratory function (spirometry), body balance, social well-being, group identification. So, our results build upon previous literature by contributing with the analysis of the effects of a singing program on these variables.

Another novel contribution of the study, due to its transdisciplinary nature, is the possibility of conjointly analyzing the benefits of the singing groups over several domains. As most studies tend to focus on the analysis of one or two domains of effects (e.g., cognitive or emotional), the present study allows for the conjoint analysis of the effects on emotional, social, cognitive and physiological domains. Therefore, the study allows for mediation and moderation analysis of the emotional, cognitive and social variables in the effects of singing groups on the physical and mental health of the individuals. This allows for a deeper understanding of the interaction between the variables in explaining the effects of singing groups intervention on active and healthy aging.

Follow-up measurement is another contribution of the study that allows for the verification of the expected gains from the program after the intervention. Will participants continue to participate in singing groups after the intervention? If yes, will the benefits continue to improve, or will the effects become stable after 4 months of intervention? If not, how long will the effects last without intervention?

Finally, including qualitative measures in the study protocol in addition to the quantitative measures allows for a deeper understanding of the effects of the singing program and of the perceived benefits of participating.

Inclusion criteria for the participants in the study is wide, as the study accepts all individuals, except those with severe impairments, incapable of following the program. We expect that individuals with a wide range of physical and/or cognitive impairments may benefit from the singing program, validating the program for the general population of older adults. In this way, the study intends to validate the singing group program as a cost-effective intervention for the promotion of the physical and mental well-being of older adults in different baseline psychological and physical conditions, and sharing a lower socioeconomic background, where cultural activities opportunities are scarcer. Results of the study are expected to be published in four scientific papers, one book memoir and presented in international conferences with scientific committees.

## Data Availability

The data sets used and or analyzed during the current study will be available from de corresponding author upon request (Iolanda Costa Galinha: igalinha@autonoma.pt; iolandag@yahoo.com).

## Abbreviations

RCT: Randomized controlled trials
EG: Experimental Group
SCM: Santa Casa da Misericordia
S-IgA: Secretory immunoglobulin A
WHOQOL, BREF & OLD: Quality of Life Scales
Dass-21: Depression, Anxiety and Stress Scale
PANAS: Positive and Negative Affect Schedule
RSES: Rosenberg Self-Esteem Scale
SWLS: Satisfaction with Life Satisfaction Scale
Social WB: Social Well-Being Scale
ULS-4: UCCLA Loneliness Scale
FISI: Social Identification Scale
FISI SG: Social Identification Scale with the Singing Group
DV: Demographic Variables
MoCA: Montreal Cognitive Assessment Test
RAVLT: Ray Auditory Verbal Learning Test
TMT-A: Trail Making Test – A
DST: Digit Symbol Test
CRP: C-Reactive Protein
ESR: Erythrocyte Sedimentation Rate
BG: Blood Glucose
BC: Blood Cholesterol
BPP: Blood Pressure and Pulse
RF: Respiratory Function measured by Spirometry
Weight: Professional Scale Weight Measurement
BB: Body Balance measured by Preferred Foot Lifted
PP: Pain Perception.
PA: Physical Activity measured by Actigraphy
SQ: Sleep Quality measured by Actigraphy
